# Overview of the Biological Activity of Anthraquinons and Flavanoids of the Plant *Rumex* Species

**DOI:** 10.3390/molecules27041204

**Published:** 2022-02-10

**Authors:** Dmitriy Berillo, Marzhan Kozhahmetova, Lina Lebedeva

**Affiliations:** 1 Department of Pharmaceutical and Toxicological Chemistry, Pharmacognosy and Botany School of Pharmacy, Asfendiyarov Kazakh National Medical University, Almaty 050040, Kazakhstan; marzhanur.7@mail.ru; 2 Department of Biotechnology, Al-Farabi Kazakh National University, Almaty 050040, Kazakhstan; 3 Department of Molecular Biology and Genetics, Al-Farabi Kazakh National University, Almaty 050040, Kazakhstan; lebedevaleena@gmail.com

**Keywords:** *Rumex confertus*, herbal medicine, anthraquinone derivatives, flavonoids, epigallocatechin gallate, epigallocatechin, emodin

## Abstract

*Rumex confertus* belongs to the genus *Rumex* and is classified as an invasive parasitic plant in agriculture. Despite other *Rumex* species being widely used in herbal medicine due to their antimicrobial, antioxidant, antitumor, and anti-inflammatory effects, there are almost no information about the potential of *Rumex* confertus for the treatment of various diseases. In this review we analyzed scientific articles revealing properties of *Rumex* plant’s substances against cancer, diabetes, pathogenic bacterial invasions, viruses, inflammation, and oxidative stress for the past 20 years. Compounds dominating in each composition of solvents for extraction were discussed, and common thin layer chromatography(TLC) and high performance liquid chromatography(HPLC) methods for efficient separation of the plant’s extract are included. Physico-chemical properties such as solubility, hydrophobicity (Log P), pKa of flavonoids, anthraquinones, and other derivatives are very important for modeling of pharmacokinetic and pharmacodynamics. An overview of clinical studies for abounded selected substances of *Rumex* species is presented.

## 1. Introduction

### 1.1. Rumex Confertus Chemical Composition and Prospects for Use

The genus Rumex is presented by approximately 200 edible and medicinal species worldwide, including *Rumex abyssinicus* Jacq, *Rumex acetosa* L., *Rumex lunaria* L., *Rumex beringensis*, *Rumex crispus* L., and others [1]. Although these plants are enriched by biologically important secondary metabolites, such as anthraquinones, flavonoids, stilbenoids, naphthalene, and phenylpropanoids, only 50 species have been used for biomedical and pharmacological investigations [1,2]. According to the results of these studies, representatives of the genus Rumex demonstrated antibacterial, bacteriostatic, antiviral, antitumor, antioxidant, and anti-inflammatory effects, and radiation protection, and might be used for treatment of various diseases [3,4,5]. Although *Rumex confertus* Willd. (Russian dock, Asiatic dock, or mossy sorrel) is characterized by the presence of anthracene derivatives and has even been included on the list of prospective herbs, it is mainly known as a severe invasive alien in Russia, Kazakhstan, China, and eastern and central Europe [6,7]. Thus, there is almost no information about its therapeutic potential, which is partly related to the incomplete study of its chemical composition.

**Figure 1:**
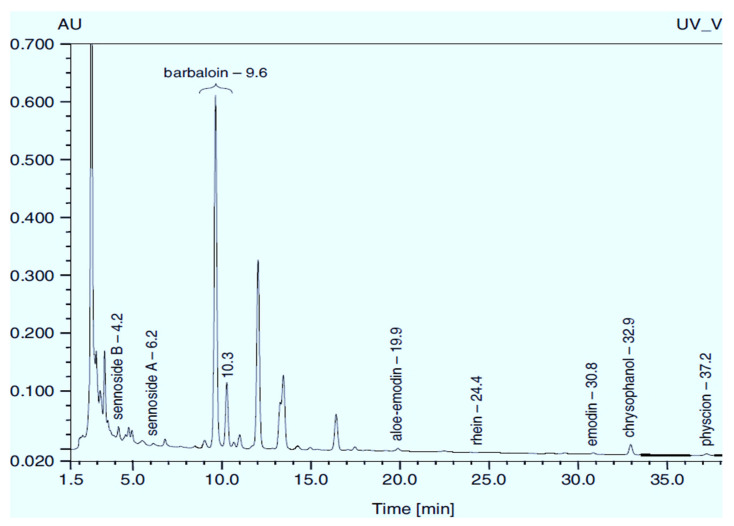
Chromatogram (RP-HPLC) of methanolic extracts from the leaves of *R. confertus* reproduced with the permission of Smolarz [8].

*Rumex confertus* Willd. is a perennial plant up to 50 cm in height, growing in meadows, forests, and forelands. The composition includes eight polyphenol anthracene derivatives such as chrysophanol, physcion, emodin, aloe-emodin, rhein, barbaloin, and sennoside A and B, which were all confirmed using HPLC analysis (Figure 1). According to the Smolarz study, the largest concentration of these compounds is detected in roots (163.42 mg/g), whereas fruits and leaves contain 0.52 mg/g and 0.67 mg/g, respectively [8]. Also a main criterion of preparation of the plant’s extract is the monitoring of content of heavy metals and other potentially toxic compounds in the plant.

The plant accumulates various compounds over the year; therefore, if researchers want to replicate published results obtaining therapeutically active extract, it is better to collect roots in spring or autumn, as stated in the protocol. To save maximum biologically active substances, it is essential to carefully wash them and dry them at room temperature. Micro and macro pharmacognostic analysis as well as conditions for extraction and TLC analysis are presented in pharmacopeia article 2.5.0052.15 [9]. Qualitative and quantitative analyses of aloe-emodin, rhein, and emodin can be also carried out using high-performance thin-layer chromatography (HPTLC) utilizing the stationary phase of a petroleum ether–ethyl acetate–formic acid (15.5:5:1, *v*/*v*) [10].

*R. confertus* Willd. total amount of anthracene derivatives can be estimated via UV spectrum at 520 nm. Dry material should contain more than 3% of 8-O-β-D-glycoside emodin. The chromatographic separation of ethanol extract on silicagel can be done using chloroform ethanol systems such as 97:1, 97:2, 97:3, 95:5, 93:7, 90:10, 85:15, 80:20, 70:30, 60:40, 50:50. Kurkin et al. reports that *R. confertus* Willd. contains the following derivatives: emodin (1,6,8-Trihydroxy-3-methyl-anthraquinone); emodin 8-O-D-glucopyranoside; chrysophanol (1,8-Dihydroxy-3-methylanthraquinone); chrysophanol (chrysophanein-8-O-D-glucopyranoside); physcion (1,8-Dihydroxy-6-methoxy-3-methylanthraquinone); nepodin (1,8-Dihydroxy-2-acetyl-3-methylnaphthalene); neposide (nepodin 8-O-D-gluco-pyranoside); and torachrysone 8-O-D-glucopyranoside (1,8-Dihydroxy-6-methoxy-2-acetyl-3-methylnaphthalene 8-O-D-glucopyranoside) [11].Litvinenko et al. investigated roots of seven *Rumex* species and suggested using 50% acetone to extract a wider range of compounds and microelements (K, Na, Ca, Mg, Fe, Zn, Ni, Mn, etc.) with efficiency of 30% from the plant. It contained tannins (22%), anthraquinones (2.8%), flavonoids (2.9%), and polyphenolic compounds (4.3%), among others [12].

Even high concentrations of these derivatives do not provoke toxic effect or mortality of animals. Guerra et al. states that oxalic acid presenting in high concentrations in *Rumex* *induratus* Boiss. & Reuter resulted in several cases of intoxication in children [13]. Crews et al. also proves that oxalic acid can cause kidney damage [14].

### 1.2. Rumex Confertus Anticancer Properties

Cancer is currently the second leading cause of death in the world. Despite progress in developing new therapeutic approaches for cancer, its occurrence increases day by day. According to WHO, almost 10 million people died from cancer in 2020 [15]. Therefore, it is necessary to discover new effective and inexpensive drugs that can improve the antitumor effects and reduce the side effects of commonly recommended chemotherapeutic drugs [16].

One of milestones of the prediction of biological activity is the hydrophilicity and hydrophobicity balance of the substance, which is represented as Log P characteristic [17]. Log P is the octanol:water partition coefficient (log *P*) of the substance. Some research revealed the structure-activity relationship correlated with Log P coefficient; therefore, we highlighted this characteristic along with pKa and solubility. For example, in the illumination of cancer cells in the presence of various substances, activities were minimal with log *P* ≤ 5, whereas it increased dramatically with log *P* in the range 5–6, and reached maximum with log *P* in the range 5.6–6.6, However, anticancer activities gradually declined with higher log *P*. The deficiency of activity of the least-lipophilic homologs of substances was explained due to their poor biodistribution characteristics, yielding insignificant tumor and plasma drug concentrations at the time of treatment with light [18]. The hydrophobicity for natural substances is a significant descriptor of bioactivity for the entire group of phenolics, for example, in decreasing order: p-courmaric acid > ferulic acid > quercetin > quercetin 3-O-glucoside > rutin. Yang et al. established correlation that the DNA cleavage activity was in a decreasing order: quercetin = rutin > p-courmaric acid > ferulic acid > quercetin 3-O-glucoside, whereas NADPH prooxidation activity was quercetin 3-O-glucoside > p-courmaric acid > rutin > quercetin > ferulic acid [19]. Such a significant difference between LD50 for rhein at oral and intraperitoneal administration is related to metabolism of the substance to rhein glucuronide and rhein sulfate as well as binding with proteins of plasma (Table 1) [20]. The value of Log P for most of compounds is in the range 2.2–4.12, and this correlates with their high antitumor activity, as was noted above. These substances are characterised by poor solubility in water, and, as expected, introducing glycoside residue leads to substantial enhance of solubility and decrease of Log P.

**Figure 2:**
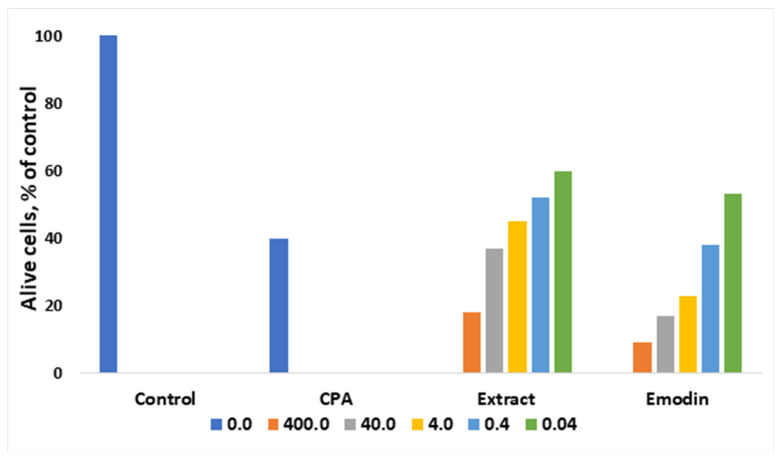
Influence of different concentrations of ethanol extract and emodin of leaves of *R. confertus* on the viability of EAC cells in vitro, adapted from Antonyan et al. [35].

The anthracene derivative emodin is known as an anticancer agent [34]. Very often, separating a complex mixture of compounds is not feasible due to cost, and therefore it is convenient to test and use plant extracts that may act in a synergetic way. Thus, as shown in Figure 2, ethanoic extract from leaves of *R. confertus* illustrated better activity against Ehrlich ascites carcinoma cells compares to the model drug emodin. Antonyan et al. studied the influence of various concentrations of ethanol extract and pure emodin obtained from *R. confertus* Willd. leaves at concentrations that varied from 0.04 to 400 μg/mL on Ehrlich ascites carcinoma (EAC) cells and non-cancerous mouse blood leukocytes (MBL). As a positive control, 1.3 μg/mL of the antitumor drug cyclophosphamide (CPA) was used [35].

The influence of various concentrations of the plant extracts *R. confertus* Willd. strongly affect the cells: the suppression of the growth of malignant EAC cells was proved by high IC_50_ values: 0.3 ± 0.04 μg/mL for ethanol extract and 40 ± 10 μg/mL for pure emodin (Figure 2). The better result of the extract usage might be related to its rich chemical composition (anthracene derivates, flavonoids, tannins, coumarins, phenol glucosides, and alkaloids). In the case of the experiments with MBL cell lines, neither pure emodin nor plant extract showed any effect at the same concentrations. MBL growth was suppressed at 25 ± 10 mg/mL (ethanol extract) and 0.2 ± 0.1 mg/mL (pure emodin), respectively.

Chun-Guang et al. also found that emodin has antitumor activity against human K562 (chronic myelocytic leukemia cell lines). Cell growth of K562 was decreased to 60% after the treatment with emodin at a concentration of 100 μmol/L, as cell cycle was terminated in phase G (0)/G (1). It was found that apoptosis-associated protein Bcl-2 decreased in a dose-dependent manner, and Bax increased after treatment with emodin. In addition, caspase-3, -8, and -9 activations have been demonstrated in vitro and in vivo. An increase in Bax along with a decrease in Bcl-2 indicates that the treatment with emodin can lead to apoptosis of K562 cells. Early apoptosis occurred in cells treated with 25, 50, and 100 µmol/L of emodin incubated for 12 h. The cell death rates in the treated groups were 8.9%, 22.9%, and 36.1%, respectively; whereas the level of apoptosis in the control group was no more than 2.1% [36].

It was observed that treatment of breast cancer cells Bcap-37 and ZR-75-30 with emodin inhibits growth and induces apoptosis. The highest growth rates were observed when cells were exposed to at least 10 μmol/L of emodin for 72 h. The highest apoptotic rate was registered at a dose of 40 mM/L. Emodin treatment inhibited Bcl-2 expression and increased caspase-3 activation, PARP, p53, and Bax levels in breast cancer cells in a concentration-dependent manner [23].

Weigelt et al. reported that although breast cancer can be classified as a local tumor, it is able to metastasize to the distant organs via blood and lymph [37]. Emodin treatment in vitro suppressed epithelial-mesenchymal transition and cancer stem cell formation by inactivation of TGF-β1-metiated interactions between tumor-associated macrophages (TAM) and breast cancer cells, and reduced cancer cells migration and metastasis formation [38].

A major factor stimulating angiogenesis in cancer tumors is vascular endothelial-derived growth factor A (VEGFA) [39]. Transcriptional factor SerRS has a potency to repress VEFGA by binding to its promoter region [40]. Zou et al. reported that emodin demonstrated the highest anti-angiogenesis activity at a concentration of 10 µM by increasing SeRS mRNA and blocking transcription of VEGFA after 48 h incubation [34]. Bai et al. also proved that emodin reduced VEGFA expression in liver cancer cells (HepG2) in vitro and inhibited tumorigenesis in BALB/c nude mice in vivo at concentrations of 1 mg/kg or 10 mg/kg [41]. Gu et al. tested emodin efficiency against colon cancer both in vitro and in vivo and found that emodin significantly suppressed migration of cancer cells and decreased the expression of VEGFA [42]. Dai et al. reported similar results after emodin treatment that decreased the levels of expression of VEGFR2, PI3K, and p-AKT in colorectal cancer HCT116 cell line [43].

Another anthracene derivative, aloe-emodin, has been reported to be an anti-cancer agent with selective activity against neuroectodermal tumors [44]. The treatment with aloe-emodin demonstrated specific dose-dependent cytotoxic effects on neuroblastoma, pPNET, and Ewing’s sarcoma cells [22]. Cell line growth of neuroectodermal tumors was specifically inhibited, and the ED50s ranged from 1 to 13 μm for neuroblastoma and Ewing sarcoma, respectively. Moreover, it induced apoptosis in human T24 bladder cancer cells via the p53-dependent apoptosis pathway [24].

Activation of mitochondrial pathways by aloe-emodin can induce the apoptosis of colon cancer cells. MTT assays were used to as measure the effect of aloe-emodin on HCT116 and NCM460 cell viability. The colony formation experiments demonstrated interesting results: in the case of increasing drug concentration and time, the cell mass extensively decreased, which shows that aloe-emodin inhibits cell proliferation in a dose-dependent way. An analysis of the assessment of cellular apoptosis showed that caspase-3 is activated and participates in the process of apoptosis induced by aloe-emodin, followed by the release of caspase-dependent members of the Bcl-2 family and cytochrome C. Using Western blotting, it was determined that the expression of the protein of the pro-apoptotic factor of the Bcl-2 Bax family increased, in contrast to the anti-apoptotic factor Bcl-2, the expression of which decreased after treatment with aloe-emodin. In addition, the results show that cytochrome C protein expression was upregulated in the cytosol but decreased in mitochondria. In the mitochondrial pathway of apoptosis, the release of cytochrome C plays a key role in the beginning of the pathway and activation of caspases [45].

Aloe-emodin, rhein, and physcion inhibit the proliferation of SCC15 cells, and the order of inhibition level are aloe-emodin > rhein > physcion; the half maximal inhibitory concentrations (IC_50_) value of aloe-emodin was 60.90 μM at 48 h of treatment. Aloe-emodin treatment resulted in a time- and dose-dependent decrease in cell viability and increased the apoptotic cell ratio. The results of Western blotting showed the expression levels of caspase-9 and caspase-3 proteins increased following aloe-emodin treatment [46].

Po-Lin Kuo et al. found that aloe-emodin inhibited cell proliferation and induced apoptosis in two human liver cancer cell lines, Hep G2 and Hep 3B. In Hep G2 cells, aloe-emodin induced p53 expression and was accompanied by induction of p21 expression, which was associated with cell cycle arrest in phase G1. In addition, aloe-emodin had a marked increase in Fas/APO1 receptor and Bax expression. On the contrary, in Hep 3B cells, in which p53 deficiency is detected, the process of inhibition of the proliferation of aloe-emodin is expressed in a p21-dependent manner, during which the cell life cycle does not stop and the number of Fas/APO1 receptors does not increase; on the contrary, it affects the increase in Bax expression and at the onset of apoptosis [31]. It was also revealed that this anthracene derivative contributes to cell death in the cell line of squamous cell carcinoma of the human lung CH27. The concentration of aloe-emodin that was lethal for 50% of CH27 cells was approximately 25 mM. Anthracene derivative-induced CH27 cell death was significant at 50 mM aloe-emodin concentration [32]. Chen et al. determined that aloe-emodin is an inhibitor of two human gastric cancer cell lines, AGS and NCI-N87. The IC_50_ value for AGS cells after 72 h of exposure was below 0.07 mM, for NCI-N87 cells it was between 0.15 and 0.19 mM, which indicates that AGS cells are more sensitive to aloe-emodin than are NCI-N87 cells [33].

It is known that telomerase enzyme plays a critical role in cancer cell proliferation. Wang et al. tested aloe-emodin on three types of breast cancer cells lines: MDA-MB-453, MDA-MB-231, and MCF-7. Aloe-emodin increased its activity in a dose-dependent manner, reducing telomerase activity to 30.5%, 26.7%, and 34.1%, respectively, at concentration of 50 μM [47].

Cheng et al. showed that aloe-emodin at concentrations of 10, 20, and 40 μM induced endoplasmic reticulum stress-dependent apoptosis in SW620 and HT29 cell lines and recommended this anthracene derivative as a potential candidate in the treatment of colorectal cancer [48]. The similar results were obtained by Shen et al. from testing in vivo the rate of caspase-dependent apoptosis induced by aloe-emodin at a concentration of 20 μM in lung cancer A549 and NCI-H1299 cell lines. The authors stated that aloe-emodin inhibited the Akt/mTOR signaling pathway [49].

Rhien suppressed the cell cycle and viability of hormone-dependent breast cancer cells (MCF-7) and hormone-independent breast cancer cells (MDA-MB-435s) under normal conditions. Rhein treatment was more effective against MDA-MB-435 cells only at a concentration of 12.5 mM, whereas at 100 mM it was more toxic for MCF-7 cells. The IC_50_ value of rhein after 48 h of incubation under normal or hypoxic conditions was 81.3 or 71.3 mM for MCF-7 cells and 52.1 or 127.3 mM for MDA-MB-435s cells [25]. Rhein inhibited proliferation of human hepatocelluar carcinoma BEL-7402 cells and increased the apoptosis rate at concentrations of 50–200 μM in 46 h. It has been reported that cell cycle S-phase was arrested via decreasing of expression of oncogene c-Myc and increasing of concentration of caspase-3 [50]. Rhein decreased the half-life of β-catenin in HepG2 cells two-fold. Moreover, it induced cell cycle arrest at S phase, which repressed proliferation of cells and further tumor growth [51]. You et al. proved that this anthraquinone induced apoptosis in HepaRG cells and cell cycle arrest at S phase by mitochondrial-mediated pathways but noted that the cytotoxicity of rhein in humans is still not clear [52].

According to the studies, Rumex contain gallic acid and flavan-3-ols, i.e., epigallocatechin gallate (EGCG), epigallocatechin (EGC), catechin and epicatechin (EC). [53,54,55] Galloylated catechins showed a stronger antiproliferative effect and apoptotic effect than non-halogenated catechins. EGCG is the most effective in the Hs578T breast cancer cell line. EGCG contains two gallate groups that can explain the highest antiproliferative effect observed in this study. EGCG, EGC, or EC can inhibit cell proliferation and modulate apoptosis. Flavan-3-ols can have a dual function, both as antioxidants and prooxidants, depending on their concentration and time of exposure to cell culture [26].

It was shown that roots of *R. confertus* contains large amounts of chrysophanol and related anthracene derivatives and are therefore considered for use in pharmaceutical practice [56]. Chrysophanol induced necrosis in J5 liver cancer cells, depending on dose and time. Cells exposed to 100 and 120 μM chrysophanol concentrations for 48 h showed S-phase arrest and cell death [27]. Analysis of cytotoxicity showed that chrysofanol nanoparticles kill prostate cancer cells compared to normal cells. Chrysophanol nanoparticles reduce histone deacetylase (HDAC) by inhibiting cell proliferation and inducing apoptosis by stopping the cell cycle in the sub-G phase. In addition, proteins associated with the cell cycle, including p27, CHK1, cyclin D1, CDK1, p-AMP activated protein kinase (AMPK), and p-protein kinase B (AKT), are regulated by chrysofanol nanoparticles to prevent human prostate cancer in vivo [57].

Caffeic acid induces apoptosis in cancer cells of the cervix, inhibiting the activity of Bcl-2, leading to the release of cytochrome c and subsequent activation of caspase-3, which indicates that caffeic acid induces apoptosis via the mitochondrial apoptotic pathway. This also suggests that caffeic acid has a strong antitumor effect [58].

### 1.3. Rumex Exctracts Anti-Inflammatory and Antioxidant Properties

Inflammation is an immediate reaction of the body to tissue injury caused by pathogens and toxic stimuli, such as biological, physical, or chemical damage. Although the inflammatory response is a protective mechanism, if it persists it can lead to multiple pathological conditions such as cancer, allergies, atherosclerosis, autoimmune diseases, and even death [59]. In mammals, heart attack results in a large amount of necrotic cardiomyocytes and an intense inflammatory response, leading to a cytokine storm [60]. It is known that cytokine storms are related to high concentrations of reactive oxygen species (ROS), which act as a chemoattractant.

Both emodin and aloe-emodin treatment inhibit oxidative stress, significantly reduce the size of infarct, and reduce the number of apoptotic cells in vivo in mice hearts due to presence of phenolic hydroxyl groups [61,62]. At a concentration of 30 mg/kg, emodin suppresses the expression of tumor necrosis factor-α (TNF-α) and the activation of NF-κB at the site of injury, preventing severe inflammation [63].

Postoperative intra-abdominal adhesions are the main complication after abdominal surgery. Emodin affects the prevention of postoperative adhesion formation compared to the control group. Such indicators of inflammation as interleukin-6 (IL-6) and transforming growth factor-β1 (TGF-β1) as well as gastrin and motilin levels were reduced after 48 h of treatment within 7 days after surgery in experimental groups in rats [64]. Emodin suppressed in vitro TNF-alpha-induced stimulation of the pro-inflammatory response. MCP-1 is a chemoattractant expressed by various types of cells, including fibroblasts. Its activation leads to the recruitment of macrophages, which, in turn, causes fibrotic reactions through their expression of TGF-β. As TNF-α is a pro-inflammatory cytokine expressed by corneal epithelial cells and macrophages, changes in its level, along with TGF-β levels, serve as indicators of the pathophysiological changes resulting from damage. Emodin at the concentration range from 1 to 5 μg/mL suppressed a dose-dependent increase in TNF-α on the expression of MCP-1 mRNA and the concentration of MCP-1 protein [65].

Lee et al. reported that emodin treatment at a concentration of 10 μM (whereas EC_50_ > 3 μM) inhibited cell death exposed by arachidonic acid and iron in vitro. Also, emodin restored mitochondria membrane that was previously destroyed by ROS [66]. Emodin also positively affected oxidative stress and apoptosis in HK-2 human renal tubular cells after hypoxia. In addition, emodin pre-treatment inhibited the process of phosphorylation of extracellular kinases [67]. Waly et al. also published results proving that emodin at a concentration of 0.5 µM significantly reduced oxidative stress in HEK 293 human kidney cells [68]. At the same time, Xie et al. reported that emodin can induce apoptosis in human colon cancer HCT116 cells by provoking oxidative stress. Emodin induced mitochondrial transmembrane potential loss, increased Bax, and decreased Bcl-2 expression. After emodin treatment, the concentration of ROS rapidly increased, which resulted in p53 overexpression [69].

Aloe-emodin dose-dependently inhibits mRNA expression induced by nitric oxide synthase (iNOS) and the production of nitric oxide (NO) at 5–40 MKM, thereby inhibiting inflammatory reactions [70].

Increased reactive oxygen species (ROS) production can also result in inflammation and pain.

### 1.4. Rumex Compounds with Antibacterial, Antimicrobial, and Antiviral Properties

Although antibiotics are widely used against bacterial infections, they have severe side effects and, in addition, bacteria are characterized by high rates of mutations and become more and more resistant to antibiotics [71]. Thus, plant extracts replace antibiotics in traditional medicine because of the absence of toxicity; moreover, they act as a complex of compounds via various pathways, providing synergistic effects of treatment and minimizing development of resistance.

Orbán-Gyapai et al. tested various fractions of Rumex species as potential agents against such bacteria as Staphylococcus epidermidis, *Staphylococcus aureus, MRSA, Bacillus subtilis, Moraxella catarrhalis, Streptococcus pyogenes, Streptococcus pneumoniae, and Escherichia coli* and proved that n-hexane and chloroform extracts from roots demonstrated significant antibacterial activity (inhibition zones > 15 mm) [72]. Ginovyan et al. recommended using acetone extracts from Rumex as the most efficient agent (76.24 µg Catechin equivalent/mg of dry weight) [73].

Ghosh et al. reported that the extract of *Rumex nepalensis* possesses some antimicrobial activity against tested microorganisms (*Staphylococcus aureus*, *Bacillus subtilis*, *Escherichia coli*, *Shigella dysenteriae,* and *Vibrio cholerae*) at all tested concentrations (200, 400, 800, and 1000 μg/disc). The inhibitory effect of the extract was observed against *Shigella dysenteriae* and comparable to the effect of chloramphenicol (10 μg/disc). Aloe-emodin is mostly responsible for the activity against *Shigella dysenteriae* [74]. Aloe-emodin, rhein, and emodin showed significant antibacterial effects on four strains of methicillin-resistant *Staphylococcus aureus* at concentrations of 2–64 μg/mL [75]. John et al. also described that the epimerized form of gallocatechin gallate had a high antagonistic effect against *E.* *coli* and *Staphylococcus aureus* accompanied by epigallo-catechin, whereas catechin gallate and (+) catechin showed lower activity against microbes [76].

Kengne et al. used ethanol and methanol as extractants for isolate active compounds from *Rumex abyssinicus*. Anthracene derivative physcion is responsible for disruption of the cytoplasmic membrane and inhibition of the microbial respiratory chain in *P. aeruginosa* and *S. flexneri* [77].

*Rumex japonicus Houtt* extract exhibited good antibacterial activity against MDR *Staph. aureus* having minimum inhibitory concentrations (MIC) ranging from 0.8 to 6.2 mg/mL and minimum bactericidal concentrations ranging from 3.1 to 12.5 mg/mL. A strong 82% inhibition of biofilm formation at sub-MIC value and eradication of biofilm at higher concentrations was observed. The motility of *Staph. aureus* was efficiently suppressed by the extract containing mainly emodin, chrysophanol, and physcion. Kim et al. stated that the extract was nontoxic to human epithelial cell lines (Caco-2 and HT-29 cell lines) at concentrations ranging from 0.1 to 0.5 mg/mL and from 0.1 to 0.75 mg/mL, respectively [78].

Noshad et al. studied antimicrobial property of methanolic extract of Rumex alveollatus L (disk diffusion agar, well diffusion agar, and minimum inhibitory/bactericidal concentration) against *Enterobacter aerogenes*, *Staphylococcus aureus, Salmonella typhi*, and *Streptococcus pyogenes*. It was stated that the extract contains 92 mg of gallic acid/g total phenols and 48.6 mg quercetin/g total flavonoids. It was evaluated that the extract was able to scavenge DPPH and ABTS free radicals by 69.6 and 80.6%, respectively, indicating good antioxidant activity. The growth of microorganisms was supressed in the presence of high concentrations of the extract, and *E. aerogenes and S. aureus* were the most resistant and sensitive bacterial species to the methanolic extract of *Rumex alveollatus* [79].

Emodin isolated from Japanese knotweed (*Polygonum cuspidatum*) at concentrations of 32 and 64 μg/mL has shown a potent inhibitory effect against *Haemophilus parasuis*, the causative agent of Glässer’s disease in pigs. At concentrations below 64 μg/mL exposure, it was found to have abnormal cell shape, plasmolysis, destroyed cell wall and membrane, and cytoplasmic vacuolation [71].

The effect of emodin on specific virulence factors of *Streptococcus mutans* (*S. mutans*) was studied in vitro and the development of caries in vivo. The growth and production of *S. mutans* acid was significantly inhibited by emodin at concentrations of 0.5–2 mg/mL. In addition, topical administration of emodin reduced the frequency and severity of carious lesions in rats [80].

It was proposed that emodin can be used as a natural alternative method for the prevention of brucellosis in animals. *Brucella (B.) abortus* is a pathogenic microorganism that results in chronic infections with multiple pathologies such as arthritis, endocarditis, and meningitis in humans, and spontaneous abortions in domestic animals. Among used concentrations of emodin, the highest non-toxic dose was 0.3 µg/mL, but higher concentrations of 3, 6, and 15 µg/mL significantly reduced survival rates of *Brucella (B.) abortus* [81].

Sennosides A, B, C, and D and rhein isolated from *Cassia pumila* Lamk demonstrated antibacterial activity against *Streptococcus pneumoniae* and *Rhizoctonia bataticola* at concentrations ranging from 100 to 400 µg/mL with the result compared to antibiotics [82]. Friedman et al. studied antimicrobial activity of seven green tea catechins and four black tea theaflavins, generally belonging to flavonoids against *Bacillus cereus* (strain RM3190). The results also show that (-)-gallocatechin-3-gallate, (-)-epigallocatechin-3-gallate, (-)-catechin-3-gallate, (-)-epicatechin-3-gallate, theaflavin-3, 3′-digallate, theaflavin-3′-gallate, and theaflavin-3-gallate showed antimicrobial activity at nanomolar concentrations; most compounds were more active than drug antibiotics such as tetracycline or vancomycin in comparable concentrations [83]. The mechanism of catechin action was described by Gradisar et al. Catechins were found to inhibit bacterial DNA gyrase by binding to the ATP binding site of a subunit of gyrase B. Epigallocatechin gallate (EGCG) was the most active in the group of four catechins, followed by epicatechin gallate (ECG) and epigallocatechin (EGC) regarding antimicrobial activity [84].

Chrysophanol (1,8-dihydroxy-3-methylanthracenedione) showed activity against Bacillus cereus, Bacillus subtilis, Staphylococcus aureus, Micrococcus kristinae, Staphylococcus epidermidis, Escherichia coli, Proteus vulgaris, Enterobacter aerogenes, and Shigella sonnei at concentrations >250 µg/mL [85].

RmlC (dTDP-6-deoxy-D-xylo-4-hexulose 3,5-epimerase) is a critical enzyme for cell wall biosynthesis in *Mycobacterium tuberculosis*. Chrysophanol is a leading inhibitor against the RmlC target with binding affinity of −9.24 kcal/mole to the RmlC active site [86].

Agarwal et al. manifested significant antifungal activity of anthraquinone derivatives such as rhein, physcion, aloe-emodin, and chrysophanol extracted from *Rheum emodi* against *Candida albicans*, *Cryptococcus neoformans*, *Trichophyton mentagrophytes*, and *Aspergillus fumigatus*. According to the results, MIC of crude MeOH extract was 250 mg/mL, whereas MIC of the pure compounds varied from 25 to 50 mg/mL, which makes them more prospective antifungal candidates [87]. Similar results on *Trichophyton mentagrophytes* (strain SM-110) have been demonstrated by Kawai et al.. Miller et al. showed antifungal activity of Barbaloin compared to lanoconazole, a commercial agent, at a minimum concentration of 75 mg/mL and 200 μg/mL in vivo and in vitro, respectively [88].

CVB3 (Coxsackie 3 virus) is the main causative agent of viral myocarditis. The study established that emodin inhibits CVB3 replication in vitro in mice. Emodin treatment at a concentration of 20 μM for 30 min inhibited mTOR signaling and activated 4EBP1 (eukaryotic initiation factor 4R-binding protein 1), resulting in suppression of translation of ribosomal protein L32. Emodin also differentially influenced several signal cascades, including Akt/mTORC1/p70(S6K) (p70 S6 kinase), ERK1/2 (extracellular-signal-regulated kinase 1/2)/p90(RSK) (p90 ribosomal S6 kinase), and Ca(2+)/calmodulin, which activate eEF2K (eukaryotic elongation factor 2 kinase) and eEF2 (eukaryotic elongation factor 2), respectively, which stop synthesis of VP1 (viral protein 1) [89].

The antiviral activity of aloe-emodin against Japanese encephalitis virus (JEV) and enterovirus 71 (EV71) was detected. The IC_50_ of aloe-emodin ranged from 0.50 mcg/mL to 1.51 mcg/mL for JEV and from 0.14 mcg/mL to 0.52 mcg/mL for EV71 [90]. Chang et al. obtained results that aloe-emodin, chrysophanol, rhein, emodin, and physcion negatively influenced JEV. The inhibitory effect of methanol extract was higher than water extract (IC_50_ = 15.04 μg/mL and IC₅₀ = 51.41 μg/mL, respectively). Separately, IC_50_ values determined by a plaque reduction assay for chrysophanol and aloe-emodin were 15.82 μg/mL and 17.39 μg/mL, respectively [91]. Derivatives of anthraquinone, such as aloe-emodin, emodin, and chrysofanol, have been stated to exhibit antiviral activity, with their inhibition mechanism and anti-influenza A activity decreasing the cytopathic effect caused by the virus and inhibition of influenza A virus replication. The IC_50_ value of aloe-emodin was less than 0.05 μg/mL [92].

The IC_50_ values were found to be 100 μg/mL and 7.3 μg/mL for abscisic acid and aloe-emodin, respectively, which indicate more potency of aloe-emodin over the abscisic acid [93].

### 1.5. Antidiabetic Activity of the Plant’s Extract Rumex

Diabetes mellitus is characterized by hyperglycemia caused by deficiency in the action or production of insulin [94]. Currently available antidiabetic agents, such as hypoglycemic drugs and insulin, have their own limitations. Natural bioactive chemicals such as flavonoids, terpenoids, alkaloids, and phenolic compounds have been described as antidiabetic agents [95].

Westermark et al. noted that amyloid peptide, which is secreted by β-cells of the pancreatic islets of Langerhans, has cytotoxic effects related to type 2 diabetes mellitus in humans and diabetes in other mammalian species [96]. Ten independent experiments showed that the number of living β-cells after three days of cultivation in the presence of 2 μM of pre-aggregated amylin significantly decreased to 20% and lower (from 40.2 × 10^5^ ± 2.6 to 7.8 × 10^5^ ± 0.8) compared with a positive control (*p* value < 0.0001).

**Figure 3:**
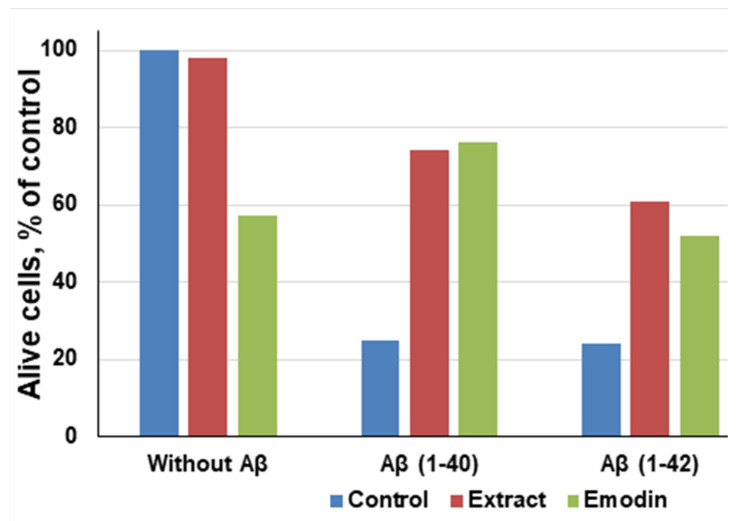
Correlation between emodin and ethanol extract of leaves of *Rumex Confertus* on the viability hippocampal cells, adapted from Antonyan et al. [35].

It was found that the cultivation of primary hippocampal cells of aggregated amyloid Aβ (1-40/42) peptides leads to their death or degradation [97,98]. Avetisyan et al. detected that the number of living cells decreased to 22.7% (from 59.8 × 10^5^ ± 4.0 to 13.6 × 10^5^ ± 1.9) in 3 days exposure in a medium containing 2 μM Aβ (1-40), pre-aggregated during 7 days. In another case, when using peptides 0.2 μM Aβ (1-42), which were aggregated under identical conditions with a duration of 5 days, the amount decreased by approximately 79.8% (from 59.8 × 10^5^ ± 4.0 to 12.1 × 10^5^ ± 1.5; *p* value < 0.0001) (Figure 3).

It has been noted that even in the absence of amyloid peptides, pure emodin at a concentration of 13.5 μg/mL decreased the number of living cells to 60%, which proves that emodin is also toxic for neuronal cells [99]. However, compared to aggregated Aβ (1-40) and Aβ (1-42) peptides, emodin is less poisonous. After the exposure of both the *R. confertus* leaves extract and pure emodin at concentrations of 3.5 μg/mL and 30 μg/mL, respectively, in the presence of Aβ (1-40), the viability of cells increased to 74%, in the presence of Aβ (1-42) increased to 61%. The IC_50_ values in the presence of Aβ (1-40) and Aβ (1-42) for the plant extract were 14.03 ± 0.13 μg/mL and for the pure emodin was 2.6 ± 0.8 μg/mL, respectively.

Anand et al. investigated in vitro the efficiency of aloe-emodin-8-O-glycoside in enhancing glucose transport by modulating the proximal and distal markers involved in glucose uptake and its conversion into glycogen even in the absence of insulin [100]. The α-glucosidase inhibition assay revealed significant activity of various samples of *R. hastatus*. Among the test samples, Rumex chloroform extract showed the highest activity of 49, 55, 61, 64, 71, and 77 at concentrations of 31.25, 62.5, 125, 250, 500, and 1000 μg/mL, respectively. The IC_50_ value was 42 μg/mL. Flavonoids and saponins also exhibited significant enzyme inhibition, with IC_50_ values of 89 and 105 μg/mL, respectively [101]. Xue at al. has shown that emodin demonstrated an activating effect on peroxisomal proliferator-activated receptor-gamma (PPARgamma) both in vitro and in vivo. Intraperitoneal injections of emodin for 3 weeks improved the symptoms in diabetic mice (serum glucose level in the experimental group was significantly lower than in the control, *p* value < 0.01), and this effect was probably associated with the regulation of the PPARγ pathway [102].

Malaguti et al. explained that rhein increases insulin sensitivity to glucose regulation in a dose-dependent manner in NOD mice [103]. Based on a rat model, it is known that 37% of rhein is excreted in urine and 53% in faeces, with a half-life of 4–10 h. Ninety-nine percent of rhein binds to plasma proteins and metabolizes primarily to rhein glucuronide and rhein sulfate. Its antidiabetic potential might be explained by suppressing the expression of dynamin-related protein 1 [20].

Rhein in cooperation with angiotensin-converting enzyme inhibitor (ACEI) can prevent the progression of diabetic nephropathy (DN) in mice suffering from type 2 diabetes. Normally DN causes kidney destruction, but after 8 weeks of rhein (150 mg/kg/day) with benazepril (10 mg/kg/day) treatment, plasma creatinine levels decreased significantly, compared with the diabetic control group by the end of the treatment period [104]. Moreover, ethanol extracts of Rhei Rhizoma containing rhein and sennosides attenuated DN, hypercholesterolemia, and platelet aggregation—the possible consequences of diabetes—in vitro via enhancing glucose uptake in 3T3-L1 adipocytes, decreasing triglyceride accumulation, and inhibiting alpha-glucoamylase activity [105].

Nepodin (Log P 3.1) isolated from Rumex roots was examined for the ability to stimulate protein kinase (AMPK) phosphorylation. In in vivo experiments, nepodin decreased the level of glucose in blood, increased the glucose intolerance in mice, and increased glucose uptake in a dose-dependent manner. In in vitro studies on L6 myotubes, it was detected that nepodin stimulated the process of 5′-adenosine monophosphate-activated and also improved AMPK phosphorylation in skeletal muscle cells [106].

As a result of the evaluation of the inhibitory potential of COX-1 and COX-2 using molecular docking and the theoretical evaluation of the absorption, distribution, metabolism, excretion, and toxicity in pharmacokinetics properties of eighteen nepodine derivatives isolated from the roots of *Rumex nepalensis*, it was found that four of them indicated enhanced inhibition of COX -2 as well as anti-inflammatory activity using rat paw edema induced by carrageenan [107].

Barbaloin (C-glucoside of aloe-emodin) also has a beneficial effect on type 2 diabetes. In mice treated by barbaloin at concentrations of 20 mg/kg and 50 mg/kg, the level of blood sugar decreased, whereas the insulin level increased [108].

Diabetic retinopathy occurs due to diabetes and is one of the most common causes of vision loss. Hyperglycemia leads to overexpression of many biological effectors, such as vascular endothelial growth factor (VEGF), which is very important for the development of diabetic retinopathy. Astragaline, the flavonoid presented in many *Rumex* species, has a beneficial effect on hyperglycemia: it helps to prevent diabetic retinopathy by reducing the excessive expression of VEGF in cultured Muller cells and weakening the effects caused by a high concentration of glucose in the blood [109].

Aldose reductase is involved in the development of secondary complications of diabetes, including cataracts, and, therefore, is the main drug target for the development of methods for treating diabetic diseases. A bioanalysis was performed based on the isolation and clarification of the structure of phloroglucinol derivative that can be isolated from *Rumex acetosa* L. 1-O-galloyl-β-D-glucose (β-glucogallin, which exhibits both selective and relatively strong inhibition (IC) 50 = 17 μM) of AKR1B1 in vitro. Molecular docking results find that this inhibitor is able to bind favorably at the active site, and β-glucogallin effectively inhibits sorbitol accumulation by 73% at 30 μM under hyperglycemia in an ex vivo organ cultivation model for lenses excised in transgenic mice with overexpression of human aldose reductase in the lens [110].

Pao-Hsuan Huang et al. established that MCF-7 cell growth was eliminated by administration of 12.5 μM aloe-emodin over 6 days, and only 50% of cells survived at 25 μM aloe-emodin treatment for 4 days. Some other nonclinical study illustrated that the IC_50_ of aloe-emodin was 46 μM for MCF-7 cells. At a concentration of 25 μM, aloe-emodin significantly inhibited proliferation in human skin epidermoid carcinoma cells compared to in noncancerous cells. Nonetheless, aloe-emodin did not significantly affect the proliferation of MDA-MB-453 ERα-negative cells. The overexpression of ERα in MCF-7 cells enlarged the sensitivity to aloe-emodin treatment. Thus, aloe-emodin has a higher cytotoxic potential to MCF-7 (ERα-positive) cells than to MDA-MB-231 (ERα-negative) cells. Moreover, even a relatively low dose of 10 μM of aloe-emodin revealed that both cell growth and ERα activation were significantly repressed by aloe-emodin in a dose-dependent manner [111]. We have summarised all available information about completed clinical trials of individual compounds that was found in Rumex plant’s in Table 2.

Rumex acetosa considerably prevents the adhesion of Porphyromonas gingivalis (P.g.) to eukaryotic host cells in vitro. The randomized placebo-controlled pilot-trial established that mouth wash by 0.8% of proanthocyanidin-enriched extract from the plant effect microbiological, clinical, and cytological parameters [117]. Supragingival debridement was followed by mouth washing (3 times per day) with the extract or placebo (control) for 7 days as adjunct to routine oral hygiene. Intergroup assessments illustrated no meaningful microbiological, cytological, and clinical differences at any timepoint. Nevertheless, significant reductions in sulcular bleeding index at day 14 (*p* = 0.003) and approximal plaque index at day 7 (*p* = 0.02) and day 14 (*p* = 0.009) were observed in the test group by intragroup comparison. There were no severe side effects. The results indicate that the plant extract mouth rinse is safe but does not seem to inhibit colonization of Porphyromonas gingivalis or improve periodontal health following supragingival debridement [117].

### 1.6. Metabolism of Plant Derivatives In Vivo

Adsorption, distribution, metabolism, and excretion are among the most important parameters, along with pharmacological activity, during drug development preclinical and clinical trials and the standardisation process of a substance. Investigation of metabolism is an extremely difficult task, which can be solved using modern physicochemical equipment such as an HPLC equipped with C18 column and mass detector and radiation isotope analysis [118].

It was observed that emodin after a single oral administration of 50 mg/kg to rats indicated urinary excretion of 18% dose in one day and up to 22% in 3 days. Metabolites evaluated in pooled urine (0–3 days) were mostly free anthraquinones (emodin and emodic acid, 16% dose), and 3% was conjugated. Thus, 48% and 68% of the dose of emodin was excreted in the faeces mostly in unchanged anthraquinone over observation time, respectively. In cannulated rats, biliary discharge achieved a maximum after 6 h and eliminated 49% of the dose after 15 h; 70% of biliary activity was in conjugated emodin. Interestingly, radioactive labelled derivative of emodin in most organs decline significantly in 72–120 h. The ^14^C activity of the substance in kidneys was estimated of 4.3 ppm after 120 h; in the mesenterium and fat tissue, an increase in ^14^C activity from 72 to 120 h was noticed [21].

The metabolism of 1,3,8-trihydroxy-6-methylanthraquinone was calculated analysing rat liver microsomes. It was found that emodin metabolites omega-hydroxyemodin and 2-hydroxyemodin were detected. It was established that formation of omega-hydroxyemodin happens in cytochrome P450 [119]. It is known that the phenolic group is sensitive to oxidation. Emodin is sensitive to prolonged exposure to light due to the phenolic functional group oxidation. Novel derivatives of aloe-emodin, with an N-heterocyclic fragment in the composition, were isolated. The structure-activity relationship (SAR) is a powerful tool to predict probability of pharmacological activity of novel substances in silico. SAR software predicted that when some groups are replaced, i.e., hydroxyethyl and benzhydryl piperazine groups, the efficiency of their application increases. Compared to the activity of aloe-emodin and corresponding benzhydryl piperazine derivatives showed a substantial inhibitory effect (IC_50_ 5.66 ± 0.47 μM) on LPS-induced nitric oxide production in macrophage cells. The emodin derivatives exhibited high bioavailability (55%). The in vivo experiments on mice with induced ulcerative colitis showed a suppressive effect on the inflammatory process, indicating the superiority of novel derivative of emodin for the design of an anti-inflammatory drug based on it [120].

## 2. Conclusions and Future Prospects

We summarised systems of solvents used for extraction of therapeutically active compounds from *Rumex*. The main focus on pharmacological activity of extracted anthracene and flavonoid derivatives obtained from the *Rumex* species is due to its wide area of growth and large quantities. A great number of studies focus on substances that can be obtained from *Rumex* species devoted to the evaluation of anticancer activity of glycosylated anthraquinones and flavonoids. *Rumex* confertus and other species contain rhein glucuronide, barbaloin, sennoside, leucocyanidin, chrysophanol, physcion, nepodin, aloe-emodin, and other derivatives that have been proven to have antibacterial, antimicrobial, antiviral, antidiabetic, anti-inflammatory, and antioxidant properties. The gap in the research related to herbal plants is the limited information about optimization of cultivation conditions at artificial conditions. Currently, special attention is given to liquid soil supplements and growth stimulants for plants along with artificial light with programmed spectral characteristics. It is known that the method of the plant cultivation, amount of sun light, and microclimate can significantly change the chemical composition of the plant extract. Therefore, there is great potential for research to optimize conditions of cultivation with a purpose to achieve the highest yield of valuable biologically active ingredients. Another area of interest is the chemical modification of extracted complex compounds for development of novel drugs. The presence of diverse functionalities in the structure of anthraquinones and flavonoid derivatives provides the possibility of modification via a linker with synthetic drugs or precursors in order to create a combinatorial library of hybrid compounds in a search for novel substances with high efficiency of treatment of serious diseases. There are not many clinical studies of prepared plant extracts that have been tested successfully in vitro. Moreover, during the clinical studies, special attention should be given to metabolites of natural compounds that might reveal unique pharmacological properties and therefore motivate researchers to explore novel substances.

## Figures and Tables

**Table 1:** Physico-chemical and toxicity data of extractive substances.

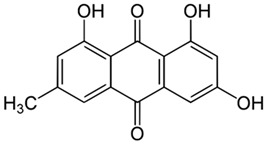	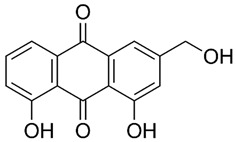		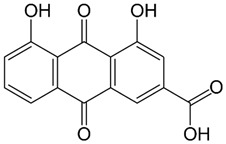
Emodin 6-methyl-1,3,8-trihydroxyanthraquinone Logp 2.7 water solubility less than 1 mg/mL at 66 °F. Solubility at 25 °C (g/100 mL of saturated solution): ether 0.140; chloroform 0.071 [16]; Ld_50_ mouse intraperitoneal 35 mg/kg [21].	Aloe-emodin 1,8-dihydroxy-3-(hydroxymethyl) anthraquinone LogP 3.25 [22], water solubility 5.5 × 10^−5^ mole/L LD_50_ mouse intraperitoneal 35 mg/kg [23].		Rhein 4,5-dihydroxy-9,10-dioxo-anthracene-2-carboxylic acid LogP 2.2 water solubility less than 1 mg/mL at 66 °F LD_50_ mouse oral 5000 mg/kg LD_50_ mouse intraperitoneal 25 mg/kg [24].
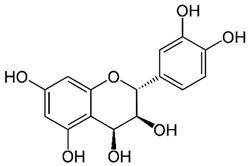	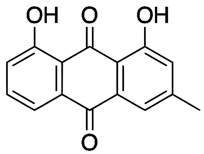		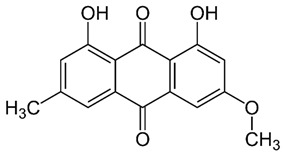
Leucocyanidin (2R,3S,4S)-2-(3,4-dihydroxyphenyl)-3,4-dihydro-2H-chromene-3,4,5,7-tetrol Water Solubility 2.22 g/L pKa 8.95 LD50 2110 mg/kg Rat Oral admin [25].	Chrysophanol or chrysophanic acid (1,8-dihydroxy-3-methylanthracene-9,10-dione) Water Solubility 0.12 g/L logP 4.12 pKa 9.14 [26].		Physcion (1,8-Dihydroxy-6-methoxy-3-methylanthraquinone) or emodin monomethyl ether Water Solubility 0.087 g/L logP 3.97 pKa 7.89 LD50 mouse intraperitoneal 10 mg/kg [27].
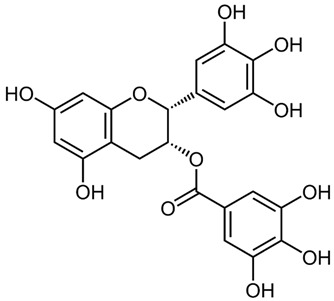		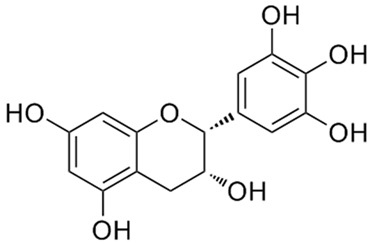	
Epigallocatechin gallate (EGCG) water solubility 32.77 mg/L at 25 °C LogP 0.639 pKa 7.75 LD_50_ 2170 mg/kg at oral administration [28].		Epigallocatechin (EGC) water solubility 0.871 mg/mL LogP 0.71 pKa 8.73 LD_50_ 1.87 mol/kg = 857 mg/kg [29].	
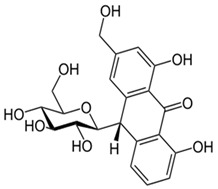		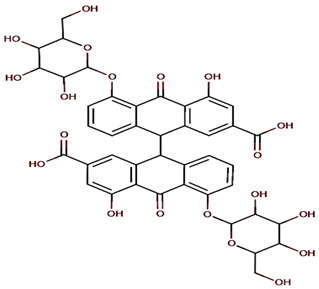	
Barbaloin or Aloin A 1,8-dihydroxy-3-(hydroxymethyl)-10-[3,4,5-trihydroxy-6-(hydroxymethyl)oxan-2-yl]-10*H*-anthracen-9-one water solubility 2.63 g/mL [30] Log P 0.42 pKa 9.51 LD50 cat oral 500 mg/kg LD50 mouse intraperitoneal 200 mg/kg [31].		Sennoside (-)-(9R*,9′R*)-5,5′-bis(β-D-gluco-pyranosyloxy)-4,4′-dihydroxy-10,10′-dioxo-9,9′,10,10′-tetrahydro-9,9′-bianthracene-2,2′-dicarboxylic acid water solubility 0.753 mg/mL LogP1.2 The LD50 value in rats was 5000 mg/kg [32] pka 3.23 [33].	

**Table 2:** Clinical trials of plant’s extractive substances.

Compound	Effect	Action	Clinical Experiment
Epigallocatechin gallate [112]	neuroprotective and neurorescue effects	modulation of cell survival and cell cycle genes	A randomised, double-blind, placebo-controlled, balanced crossover study the effects of 135 mg and 270 mg pure EGCG in 24 healthy, young adults (18–35)
Emodin [113]	antiproliferative effect	inhibits the activity of the 26S proteasome in vitro and in vivo	HEK293A-luciferase-cODC cells were seeded in 96-well plates and treated in the presence of emodin
Sennoside [114]	laxative	increasing cyclic 3′,5′-adenosine monophosphate it alter permeability of cell walls in the colon, which regulates the process of active ion secretion	Participants aged 10–18 years were randomly assigned to receive either PEG 60 mL/kg/day or PEG 30 mL/kg/day plus oral bisacodyl 10–15 mg/day or sennosides 2 mg/kg/day for 2 days prior to the colonoscopy
Aloe-emodin [115]	Triptolide Woldifii for Autosomal Dominant Polycystic Kidney Disease	MRI calculated kidney volume, eGFR [Time Frame: Every 3–6 months] End-stage kidney disease (ESRD) [Time Frame: every 2 months]	Interventional 300 participants from 15 to 70 years (child, adult, older adult) Randomized administration of Emodin (Frangula emodin, Frangulic acid) at concentration 100 mg/d
Epigallocatechin anticancer drug [116]	To determine whether the daily consumption of decaffeinated green tea catechins (Polyphenon E^®^) for 1 year reduces the rate of progression to prostate cancer (PCa) in men diagnosed with HGPIN or ASAP	Study of Polyphenon E in Men with High-grade Prostatic Intraepithelial Neoplasia	240 (120 men/arm) men 30 Years to 80 Years (adult, older adult) diagnosed with the prostate condition HGPIN or ASAP with a capsule form of standardized green tea extract called Polyphenon E (200 mg epigallocatechin gallate (EGCG) twice a day) or placebo for a 12-month period and see if it can prevent progression of the prostate condition to prostate cancer. Investigators wanted to see if Polyphenon E reduces lower urinary tract symptoms and if this can be taken safely over one year
